# A Quality Improvement Project to Assess and Improve the Recognition of Compartment Syndrome by Nurses in the Orthopedic Department

**DOI:** 10.7759/cureus.11179

**Published:** 2020-10-26

**Authors:** Calum Robertson, James Baggott, James Duncan

**Affiliations:** 1 Trauma and Orthopaedics, Buckinghamshire Healthcare NHS Trust, Aylesbury, GBR; 2 Trauma and Orthopaedics, Oxford University Hopsitals NHS Trust, Oxford, GBR

**Keywords:** compartment syndrome, trauma and orthopedics, quality improvement, education, recognition, nursing

## Abstract

Delayed recognition of compartment syndrome can result in devastating consequences such as the need for amputation or even death. Nurses are at the frontline of patient care in the orthopedic department and it is essential that they have a high index of suspicion for compartment syndrome. In this publication we describe an easily replicable project to assess and improve the understanding of the condition by nurses on trauma wards. Our project involved a questionnaire to assess the ability of nurses to recognise the key clinical features of compartment syndrome. This initial questionnaire was followed by a one-week teaching programme within the department, after which the questionnaire was repeated. Our results demonstrate that nursing staff place a disproportionate emphasis on neurovascular compromise in recognising the condition. Only just over one half (11/21) could correctly identify 'pain out of proportion to the associated injury' as the key clinical feature. Unlike pain, neurovascular compromise is a late feature of compartment syndrome and overstating its importance may potentially contribute to delayed diagnosis. Our targeted educational week dramatically improved the number of correct responses. One month after the teaching week, 83% (19/23) of nurses correctly identified pain as the most important feature in compartment syndrome. We hope that improved knowledge of compartment syndrome by nurses will help to reduce delayed recognition and adverse outcomes.

## Introduction

Acute limb compartment syndrome is a surgical emergency that results from raised pressure within a closed myofascial compartment which leads to local tissue ischaemia. It is predominantly a clinical diagnosis based on the recognition of pain that is out of proportion to the initial injury [1]. Intra-compartmental pressure testing can be used to aid diagnosis and is particularly useful in unconscious/intubated patients. Late features of the condition include absent pulses in the affected limb and neurological sequelae such as reduced sensation and motor control. In order to prevent complications such as acute limb ischemia, urgent surgical exploration should be undertaken in any patient where there is a suspicion of compartment syndrome. Since it is a life and limb threatening condition, it is essential that the features of compartment syndrome are recognised by all staff working in an acute trauma and orthopedic department [2]. Despite this, cases of missed compartment syndrome still occur [3], and are a frequent source of litigation in orthopedic surgery [4]. 

One factor that could contribute to a delayed diagnosis of compartment syndrome may be a disproportionate emphasis on neurovascular compromise as the hallmark of the condition when in fact pain is the main clinical finding [5]. Indeed, there are many case reports where post-operative regional analgesia has masked the initial symptoms of compartment syndrome and has resulted in a delayed diagnosis [6]. Since nurses are at the front line of looking after these patients, we wanted to assess the confidence and ability of the nursing staff at our local district hospital to recognise the clinical features of compartment syndrome. We are looking in particular to assess whether the nursing staff can recognise that pain is a more important clinical finding than neurovascular compromise. Secondly, we organised a targeted, innovative educational programme to assess whether this would improve awareness of the condition and improve the nurses' confidence to be able to recognise it. In this publication we outline the approach of our project, which may be used as a template in other hospitals.

## Materials and methods

The first part of the project assesses the ability of nursing staff to understand the clinical features of compartment syndrome. To do this, we designed a questionnaire based upon the British Orthopedic Association Standards for Trauma [7]. Questionnaires were distributed in person to all nursing staff over a period of one week - they answered the questions anonymously and were asked not to confer with colleagues or use resources to help them answer the questions.

Once the data was collected, we developed a ‘Compartment Syndrome Recognition Week’. This consisted of a daily 20-minute teaching session delivered by doctors at the nursing morning handover, as well as placing numerous information posters throughout the department. The teaching sessions covered the same content and were repeated daily in order to cover the shift pattern for all the nurses working in the department. An example of the slides used in the teaching PowerPoint and the posters placed throughout the department can be found in the appendices.

One month after the education week, a second round of questionnaires was given to all the nursing staff at handover. They were asked to complete the questionnaire in the same manner as previously. For each question, the number of correct responses before and after teaching were compared using a Chi-squared (X^2^) test. 

## Results

**Figure 1:**
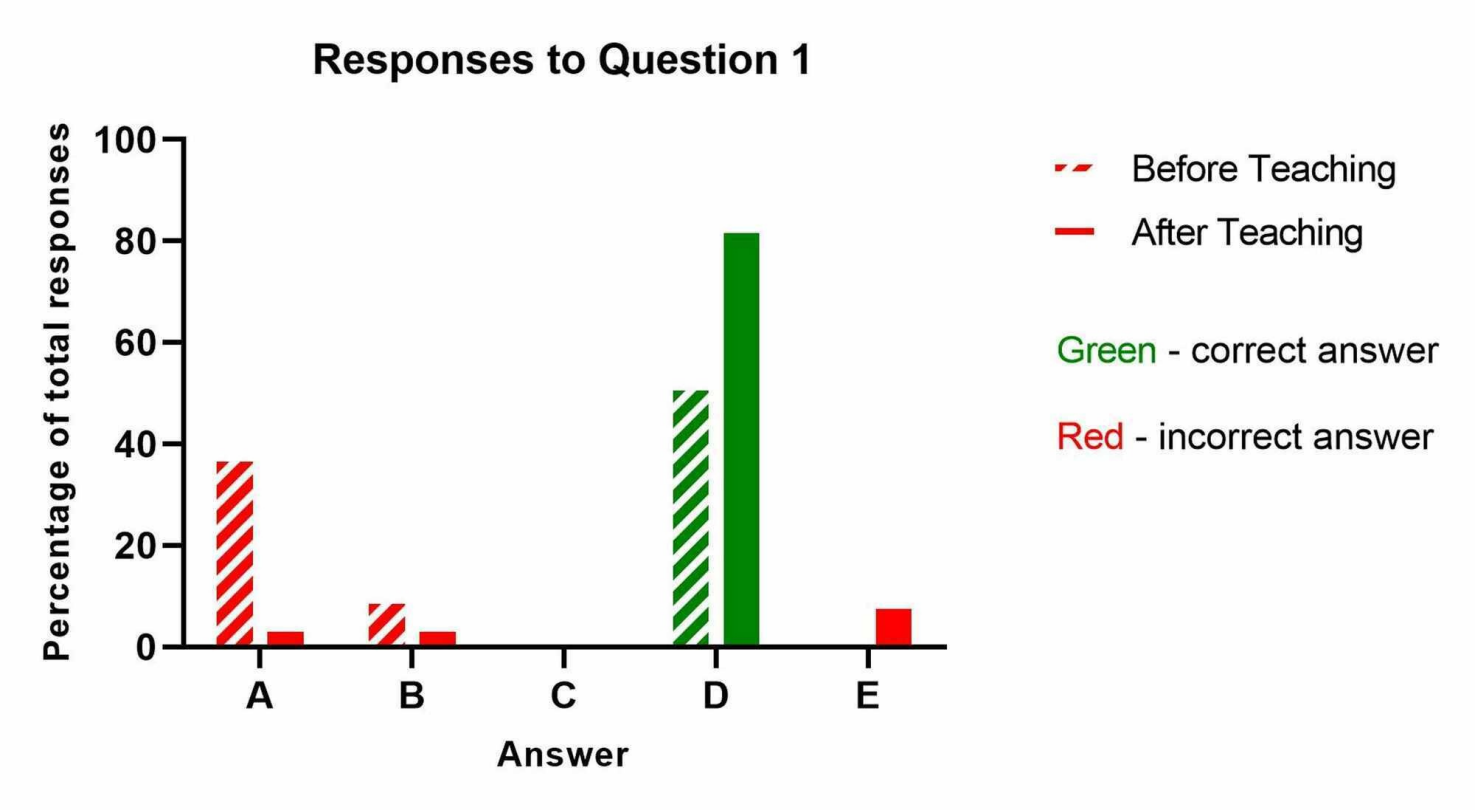
What is the single most important clinical finding in compartment syndrome?

In total, 21 responses were obtained from nursing staff prior to the targeted education week and 23 responses were obtained after. The results from question one ('What is the single most important clinical finding in compartment syndrome?') are shown below (Table 1 and Figure 1). The proportion of nurses who correctly identified ‘pain out of proportion to the associated injury’ as the main clinical feature increased from 52% to 83%. In contrast, whilst nurses initially seemed to overstate the importance of neurovascular observations in the recognition of compartment syndrome (38% and 10% of nurses choosing the absence of pulses and inability to move the affected limb respectively as the most important clinical finding), this was no longer the case after teaching (with each response getting 4.5% of responses in the second questionnaire). When comparing the total number of correct responses before and after teaching using a Chi-squared test, there was a significant improvement in the nurses responses after teaching (p=0.03).

**Table 1:** What is the single most important clinical finding in compartment syndrome?

	Before Teaching	After Teaching
A: Lack of pulses in affected limb	8	1
B: Unable to move extremities in the affected limb	2	1
C: Cold extremities in the affected limb	0	0
D: Pain out of proportion to the associated injury - CORRECT ANSWER	11	19
E: Change in sensation in the affected limb	0	2

**Figure 2:**
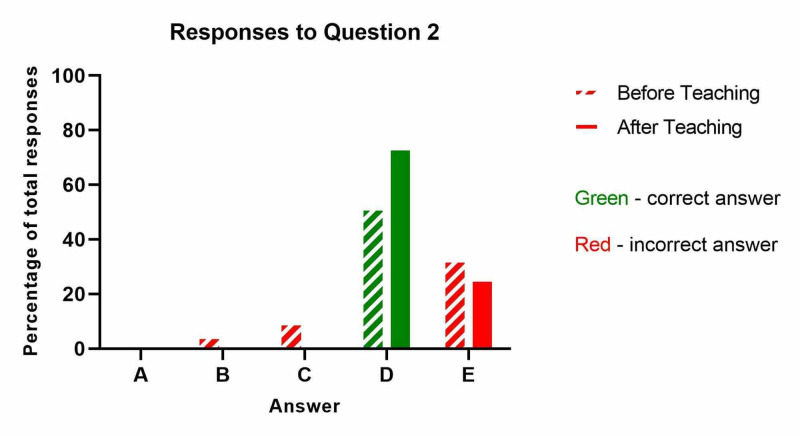
What is the minimum frequency of nursing limb observations for patients at high risk of compartment syndrome?

The results from question two ('What is the minimum frequency of nursing limb observations for patients at high risk of compartment syndrome?') are shown below (Table 2 and Figure 2). Whilst there was an increase in the proportion of correct answers after teaching from 52% to 72%, this did not meet the threshold for significance (p=0.14). 

**Table 2:** What is the minimum frequency of nursing limb observations for patients at high risk of compartment syndrome?

	Before Teaching	After Teaching
A: 8 hourly	0	0
B: 4 hourly	1	0
C: 2 hourly	2	0
D: 1 hourly - CORRECT ANSWER	11	17
E: Every 30 minutes	7	6

**Figure 3:**
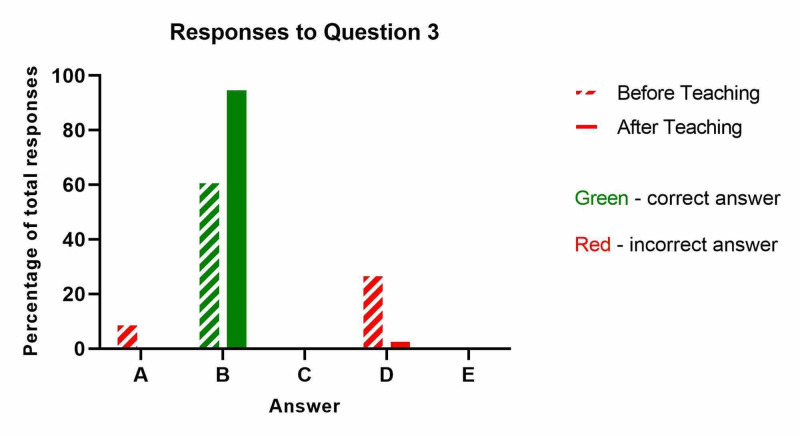
What is the first management step in suspected compartment syndrome?

The results from question three ('What is the first management step in suspected compartment syndrome?') are shown below (Table 3 and Figure 3). The proportion of nurses who correctly identified the initial management step in compartment syndrome increased from 62% to 96%, and this improvement was statistically significant (p=0.005).

**Table 3:** What is the first management step in suspected compartment syndrome?

	Before Teaching	After Teaching
A: Strong analgesia (e.g. PCA)	2	0
B: Release all circumferential dressings to skin and elevate the affected limb to heart level. Reassess within 30 minutes. - CORRECT ANSWER	13	22
C: IV fluids	0	0
D: Urgent decompressive surgery	6	1
E: IV antibiotics	0	0

**Figure 4:**
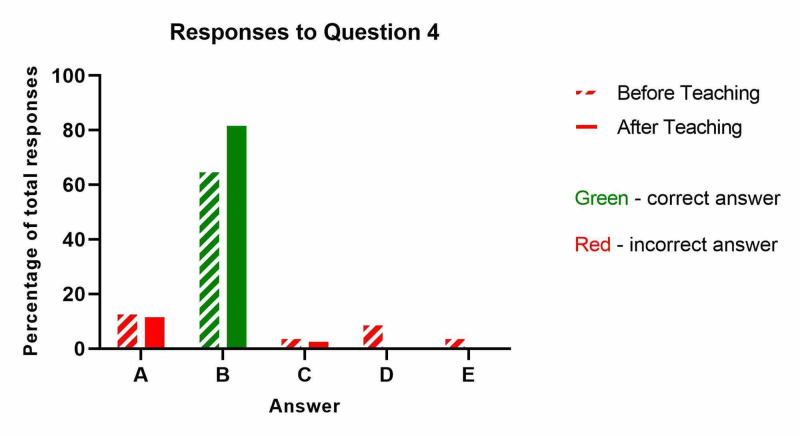
Which of the following is not a risk factor for compartment syndrome?

The results from question four ('Which of the following is not a risk factor for compartment syndrome?') are shown below (Table 4 and Figure 4). Whilst the proportion of nurses who correctly identified that old age was not a risk factor for compartment syndrome increased from 66% to 88%, this did not meet the threshold for significance (p=0.22).

**Table 4:** Which of the following is not a risk factor for compartment syndrome?

	Before Teaching	After Teaching
A: Long bone fracture (e.g. anterior tibial injury)	3	3
B: Old age - CORRECT ANSWER	14	19
C: Prolonged surgery for a limb injury	1	1
D: Crush injury	2	0
E: Injured limb that has been placed in a cast	1	0

**Figure 5:**
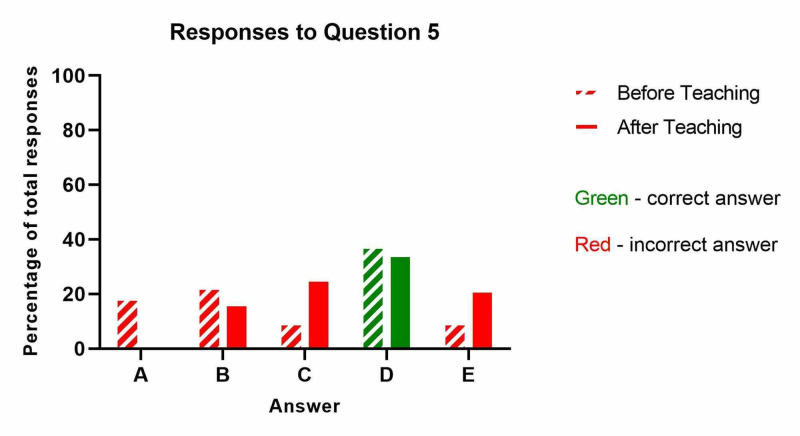
How soon should decompressive surgery occur for suspected compartment syndrome?

The results from question five ('How soon should decompressive surgery occur for suspected compartment syndrome?') are shown below (Table 5 and Figure 5). Surprisingly, the number of correct answers decreased slightly after teaching, however this was not significant (p=0.82). 

**Table 5:** How soon should decompressive surgery occur for suspected compartment syndrome?

	Before Teaching	After Teaching
A: Within 12 hours	4	0
B: Within 6 hours	5	4
C: Within 2 hours	2	6
D: Within 1 hour - CORRECT ANSWER	8	8
E: Within 30 minutes	2	5

We also collected data from the feedback received for the teaching sessions which were delivered at nursing handover. On a scale of 0-10, the teaching received a mean score of 9.7 for delivery, 9.9 for usefulness, and 9.4 for improving confidence at being able to recognize and escalate a possible compartment syndrome.

## Discussion

As a result of our quality improvement project, there was a significant increase in the proportion of nurses who could correctly identify pain as the most important clinical finding in compartment syndrome. This demonstrates the effectiveness of a targeted education week at improving the ability and confidence of nurses to recognize important clinical conditions. This type of intervention is particularly critical where a condition is not routinely seen (we only see around four cases a year in our district hospital), but nonetheless extremely important to identify, such as compartment syndrome. Ideally, a more accurate measure of whether targeted educational interventions were beneficial would be to perform a prospective data collection study looking at the proportion of ‘missed’ compartment syndromes (or late diagnoses) over a period of time and compare this to data before the ‘awareness week’. Given the low frequency of compartment syndrome, such a study would have to be multi-centre and would be logistically challenging. In this instance, we believe that a targeted questionnaire focusing on the pertinent clinical features of compartment syndrome is an effective substitute to see how efficient a department would be in the early recognition of the syndrome.

What this study does not however tell us is the durability of any benefits from this targeted educational intervention. Whilst we can conclude that this educational week appears to have a highly beneficial short-term effect, it is difficult to assess whether this would make a difference over the long term. There are two principal concerns - one is that staff would not retain any knowledge gained over a long period of time (especially if the condition is not often encountered), and the second is that this knowledge may dissolve with a high turnover of nursing staff. In order to assess the duration of the beneficial effect from this targeted education intervention, it may be required to repeat the questionnaire in one year’s time. Any future study would be able to compare the pre-teaching answers to the answers post-teaching from this project. If there is a significant deterioration over this time period, then the educational intervention may need to be repeated at a higher frequency (e.g. every six months) to be effective in the long-term. We hope that posters will help to mitigate this.

## Conclusions

Firstly, this project demonstrates that nursing staff may overstate the importance of neurovascular compromise in acute limb compartment syndrome. Given that pain is the key clinical hallmark of the syndrome and neurovascular compromise can be a late feature, this may lead to a delayed diagnosis. Secondly, our ‘Compartment Syndrome Recognition Week’ significantly improved the confidence and ability of nursing staff to understand the syndrome. This demonstrates just how effective a targeted educational intervention can be at improving the recognition of uncommon yet important conditions. We hope that our project can be used as a template for other orthopedic departments, and that a similar approach can be used for other important conditions. 

## Figures and Tables

**Figure 6:**
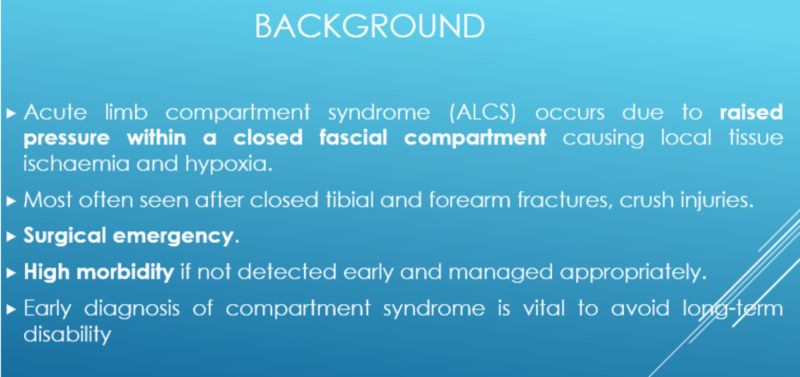
Example of a slide taken from the educational powerpoint presentation used for teaching in the Compartment Syndrome Recognition Week.

**Figure 7:**
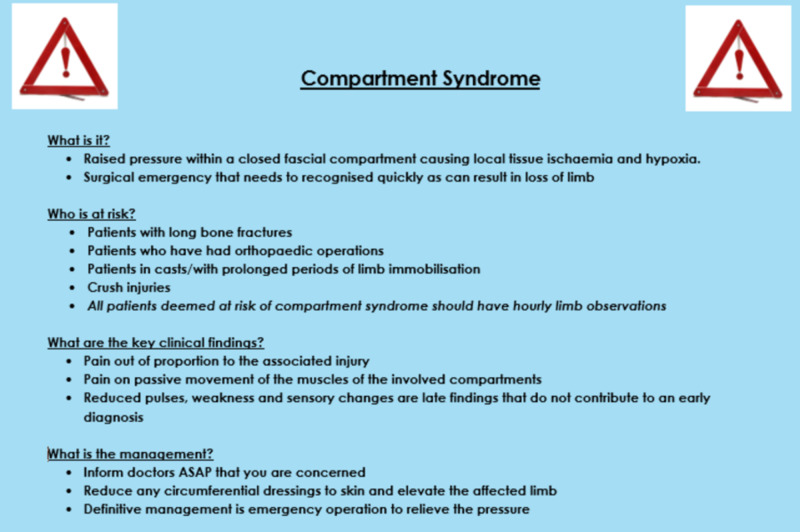
Example of one of the posters displayed throughout the orthopedic department during Compartment Syndrome Recognition Week
